# Effects of empagliflozin on nondiabetic salt-sensitive hypertension in uninephrectomized rats

**DOI:** 10.1038/s41440-019-0326-3

**Published:** 2019-09-19

**Authors:** Sua Kim, Chor Ho Jo, Gheun-Ho Kim

**Affiliations:** 1 Institute of Biomedical Science, Hanyang University College of Medicine, Seoul, Korea; 2 Department of Internal Medicine, Hanyang University College of Medicine, Seoul, Korea

**Keywords:** Empagliflozin, Hypoxia-inducible factor-1, Inflammation, Salt-sensitive hypertension

## Abstract

Impaired pressure natriuresis (PN) underlies salt-sensitive hypertension, and renal inflammation and hypoxia-inducible factor-1 (HIF-1) have been implicated in the modulation of systemic hypertension. Although sodium–glucose cotransporter-2 (SGLT2) inhibitors were reported to lower blood pressure (BP) in type 2 diabetes mellitus, whether they have a role in nondiabetic hypertensive kidney diseases is unclear. The present study was undertaken to investigate whether nondiabetic salt-sensitive hypertension and accompanying renal inflammation are ameliorated by SGLT2 inhibition. Male Sprague-Dawley rats were randomly divided into three groups: sham controls (SCs), uninephrectomized controls (UCs), and empagliflozin-treated rats (ETs). All rats were fed a rodent diet with 8% NaCl throughout the study period. Empagliflozin was orally administered for 3 weeks after uninephrectomy. Systolic blood pressure was recorded weekly, and kidneys were harvested for immunoblotting, immunohistochemistry, and quantitative PCR analysis at the end of the animal experiment. Systolic BP was significantly decreased in ETs that were orally given empagliflozin for 3 weeks after uninephrectomy. Although ETs did not show any increase in weekly measured urine sodium, the right-shifted PN relationship in UCs was improved by empagliflozin treatment. The expression of HIF-1α was increased in the renal outer medulla of ETs. Consistent with this, HIF prolyl-hydroxylase-2 protein and mRNA were decreased in ETs. The abundance of CD3 and ED-1 immunostaining in UCs was reduced by empagliflozin treatment. The increased IL-1ß, gp91phox, and NOX4 mRNA levels in UCs were also reversed. Empagliflozin restored impaired PN in nondiabetic hypertensive kidney disease in association with increased renal medullary expression of HIF-1α and amelioration of renal inflammation.

## Introduction

Recent clinical trials have demonstrated that sodium–glucose cotransporter-2 (SGLT2) inhibition may improve cardiovascular and renal outcomes in type 2 diabetes [1–3], probably via lowering blood pressure and restoring tubuloglomerular feedback [4]. Many nondiabetic kidney diseases have accompanying systemic and glomerular hypertension, but whether afflicted patients may see benefits with the use of SGLT2 inhibitors is unclear.

The pressure–natriuresis (PN) relationship explains the central role of the kidney in the pathogenesis of hypertension [5]. In chronic kidney disease (CKD), the ability to increase urinary sodium chloride (NaCl) excretion in response to high salt intake is diminished. Thus, patients with CKD are frequently plagued by volume overload and often have their PN curve shifted downward and to the right, signifying salt sensitivity [6]. Previous studies have shown that renal inflammation plays a role in salt-sensitive hypertension by impairing PN [7, 8] and that oxidative stress in the kidney may decrease medullary blood flow, impair sodium excretion, and increase blood pressure [9].

Hypoxia inducible factor (HIF)-1α is mainly localized in the renal medulla, and its expression is inhibited by HIF prolyl-hydroxylase-2 (PHD2) [10]. The renal medullary expression level of HIF-1α may play a role in salt-sensitive hypertension because of its promoting effect on natriuresis [11]. In Dahl salt-sensitive rats, high NaCl intake did not activate HIF-1α in the renal medulla or its target genes (i.e., heme oxygenase-1, nitric oxide synthase-2, and cyclooxygenase-2) to maintain the PN relationship [12]. However, the effect of SGLT2 inhibitors on PN has not been explored in nondiabetic kidney disease to date.

This study was undertaken to investigate whether nondiabetic salt-sensitive hypertension and its accompanying renal inflammation are ameliorated by SGLT2 inhibition. For this aim, the animal model of salt-sensitive hypertension was induced by uninephrectomy and 8% NaCl intake in male Sprague-Dawley rats. Empagliflozin (20 mg/kg/d) was orally administered for 3 weeks after uninephrectomized rats were stabilized over 2 weeks. This animal model of salt-sensitive hypertension was modified from that of Guyton’s study in which salt sensitivity was induced in dogs with renal mass reduction [13].

## Materials and methods

### Animal experiments

Pathogen-free male Sprague-Dawley rats weighing 200–220 g (Orient Bio Inc., Seongnam, Korea) were fed a rodent diet with 8% NaCl throughout the study period. They were randomly divided into three groups: control rats with sham surgery (SCs), control rats with uninephrectomy (UCs), and empagliflozin-treated rats with uninephrectomy (ETs). In the ETs, empagliflozin (20 mg/kg/d) was orally administered for 3 weeks after the uninephrectomized rats were stabilized over 2 weeks. Unanesthetized systolic blood pressure (SBP) was measured using the tail-cuff method (BP-98A; Softron, Tokyo, Japan). Before the SBP measurement, rats were restrained by acrylic holders and placed in a chamber at 29–32 °C for 30 min. Each measurement was obtained by averaging at least three trials from each rat. The order of these measurements was randomized, and only one person performed the testing, eliminating between-observer bias. The kidneys were harvested at the end of the animal experiment for immunoblot analysis, immunohistochemistry, and quantitative polymerase chain reaction (qPCR). The experimental protocol was approved by the Institutional Animal Care and Use Committee of Hanyang University (No. 2017-0068A).

### Biochemical analysis

The rats were housed in individual metabolic cages (Tecniplast, Buguggiate, Italy) for urine collection. Plasma and urine parameters were measured weekly, and blood samples were collected from tail veins. Biochemical analyses were performed by DKKorea Inc. (Seoul, Korea) with an automated analyzer (AU680; Beckman Coulter, Brea, CA, USA) using the Jaffe method for creatinine, ion-selective electrode method for sodium, biuret method for protein, enzymatic method for glucose, and bromocresol green method for albumin. Urine osmolality was determined by an Advanced 2020 Osmometer (Advanced Instruments, Norwood, MA, USA) using the freezing-sedimentation method.

### Immunoblot analysis

Rat kidneys were dissected to obtain outer medullary tissue and then placed in ice-cold isolation solution and homogenized using a tissue homogenizer [14]. Protein samples were separated by sodium dodecyl sulfate–polyacrylamide gel electrophoresis using 10% gels for HIF-1ɑ and 12% gels for PHD2 protein expression. Proteins were transferred electrophoretically from unstained gels to nitrocellulose membranes (Bio-Rad, Hercules, CA, USA). After blocking with 5% skim milk in PBS-T (80 mM Na_2_HPO_4_, 20 mM NaH_2_PO_4_, 100 mM NaCl, 0.1% Tween-20, pH 7.5) for 1 h, membranes were probed overnight at 4 °C with the following primary antibodies: mouse monoclonal anti-HIF-1α (Novus Biologicals, Catalog no. NB100-105, Littleton, CO, USA), rabbit polyclonal anti-PHD2 (Novus Biologicals, no. NB100-2219), and mouse monoclonal anti-β-actin (Sigma-Aldrich, Catalog no. A5441, St. Louis, MO, USA). The secondary antibodies were goat anti-mouse (Catalog no. 115-035-003) and goat anti-rabbit (Catalog no. 111-035-003) conjugated to horseradish peroxidase (Jackson ImmunoResearch, West Grove, PA, USA). The sites of antibody–antigen reaction were viewed using enhanced chemiluminescence (GenDEPOT, Barker, TX, USA), and the band densities on immunoblots were quantified by densitometry using a laser scanner and Quantity One software (Basic version 4.6.9; Bio-Rad).

### Immunohistochemistry

Immunohistochemical staining for HIF-1α, CD3, and ED1 was performed on formalin-fixed, paraffin-embedded sections using the microwave antigen retrieval method. The primary antibodies were mouse monoclonal anti-HIF-1α (Novus Biologicals, Catalog no. NB100-105, Littleton, CO, USA), rabbit monoclonal anti-CD3 (Abcam Inc., Catalog no. ab5690, Cambridge, MA, USA), and mouse monoclonal anti-rat ED1 (Serotec, Catalog no. MCA341R, Oxford, UK). For CD3 and ED1 immunostaining, a point-counting technique was used to calculate the number of positively stained interstitial cells in at least 20 consecutive high-power fields.

### Quantitative polymerase chain reaction (qPCR) analysis

Total RNA was isolated from the rat whole kidney with TRIzol® Reagent (Life Technologies, Carlsbad, CA, USA). RNA was quantified by spectrophotometry, and complementary DNA (cDNA) synthesis was performed using 3 μg of RNA with SuperScript® III Reverse Transcriptase (Life Technologies). For qPCR, 100 ng of cDNA served as a template for PCR amplification using the Brilliant SYBR Green QPCR master mix, according to the manufacturer’s instructions (FastStart DNA Master SYBR Green I; Roche Molecular Biochemicals, Mannheim, Germany). Serial dilutions (1 ng/μL to 1 fg/μL) of cDNA were used as a template to generate a standard curve. Nested primers were used to amplify the standard and kidney cDNA samples (Table 1). The standard and unknown samples were amplified in duplicate in 96-well plates. The thermal profile of the LightCycler® Instrument (Roche Molecular Biochemicals) was optimized with an initial denaturation for 10 min at 95 °C and 45 amplification cycles, each consisting of 10 s at 95 °C, 10 s at 60 °C, and 10 s at 72 °C. The comparative Ct method was used to determine the relative amounts of target-mRNA levels, expressed for each sample as a percentage of the GAPDH mRNA level. Ct ratios were analyzed using LightCycler® software (version 4.05). Specificity was verified by post-run melting-curve analysis.

**Table 1:** Primer sequences for qPCR

Gene	Forward (F) and reverse (R) primer sequences	PCR product (bp)	GenBank Accession No.
PHD2	F 5′-CTGGGACGCCAAGGTGA-3′ R 5′-CAATGTCAGCAAACTGG-3′	71	XM_017590462.1
HO-1	F 5-TCTATCGTGCTCGCATGAAC-3′ R 5′-CAGCTCCTCAAACAGCTCAA-3′	109	NM_012580.2
ET-1	F 5′-TCTACTTCTGCCACCTGGACAT-3′ R 5′-GAAGGGCTTCCTAGTCCATACG-3′	76	XM_017600453.1
eNOS	F 5′-CACACTGCTAGAGGTGCTGGAA-3′ R 5′-TGCTGAGCTGACAGAGTAGTA-3′	109	XM_006235872.1
COX-2	F 5′-ACCAACGCTGCCACAACT-3′ R 5′-GGTTGGAACAGCAAGGATTT-3′	128	S67722.1
VEGF	F 5′-CTTCCTATTCCCCTCTTAAATCGTG-3′ R 5′-CTACCTCTTTCCTCTGCTGATTTCC-3′	102	NM_001287114.1
NLRP3	F 5′-CAGACCTCCAAGACCACGACTG-3′ R 5′-CATCCGCAGCCAATGAACAGAG-3′	128	NM_001191642.1
CASP1	F 5′-TGCCTGGTCTTGTGACTTGGAG-3′ R 5′-ATGTCCTGGGAAGAGGTAGAAACG-3′	134	NM_012762.2
ASC	F 5′-TTATGGAAGAGTCTGGAGCTGTGG-3′ R 5′-AATGAGTGCTTGCCTGTGTTGG-3′	102	NM_172322.1
IL-1β	F 5′-GAGGCTGACAGACCCCAAAAGAT-3′ R 5′-GCACGAGGCATTTTTGTTGTTCA-3′	339	NM_031512
MCP-1	F 5′-GGTCTCTGTCACGCTTCTG-3′ R 5′-TTCTCCAGCCGACTCATTG-3′	145	NM_031530
RANTES	F 5′-CACCTGCCTCCCCATATG-3′ R 5′-TTCCTTCGAGTGACAAAGACG-3′	146	NM_031116
NOX1	F 5′-GGAGTTGCAGGAGTCCTCATTTT-3′ R 5′-TTCTGCCGGGAGCGATAA-3′	119	NM_053683.1
gp91^phox^	F 5′- AAAGGAGTGCCCAGTACCAAAGT-3′ R 5′-TACAGGAACATGGGACCCACTAT-3′	79	AF298656
p47^phox^	F 5′-ACGCTCACCGAGTACTTCAACA-3′ R 5′-TCATCGGGCCGCACTTT-3′	96	AY029167
P67^phox^	F 5′-GCTTCGGAACATGGTGTCTAAGA-3′ R 5′-AGAGTCAGGCAGTAGTTTTTCACTTG-3′	220	AB002664
NOX4	F 5′-AGAATGAGGATCCCAGAAAGCTT-3′ R 5′-ATGAGGAACAATACCACCACCAT-3′	89	NM_053524.1
CuZn-SOD	F 5′-TGTGTCCATTGAAGATCGTGTGA-3′ R 5′-TCTTGTTTCTCGTGGACCACC-3′	85	NM_017050
Mn-SOD	F 5′-TTAACGCGCAGATCATGCA-3′ R 5′-CCTCGGTGACGTTCAGATTGT-3′	76	NM_017051
GAPDH	F 5′-AGACAGCCGCATCTTCTTGT-3′ R 5′-CTTGCCGTGGGTAGAGTCAT-3′	200	XM_017593963

*qPCR* quantitative polymerase chain reaction, *PHD2* prolyl-hydroxylase-2, *HO-1* heme oxygenase-1, *ET-1* endothelin-1, *eNOS* endothelial nitric oxide synthase, *COX-2* cyclooxyganase-2, *VEGF* vascular endothelial growth factor, *NLRP3* NOD-like receptor family, pyrin domain-containing 3, *CASP1* caspase-1, *ASC* apoptosis-associated speck-like protein containing a caspase activation and recruitment domain, *IL* interleukin, *MCP1* monocyte chemotactic protein-1, *RANTES* regulated on activation, normal T-cell expressed and secreted, *NOX* nicotinamide adenine dinucleotide phosphate oxidase, *CuZn-SOD* intracellular superoxide dismutase, *Mn-SOD* mitochondrial SOD, *GAPDH* glyceraldehyde-3-phosphate dehydrogenase

### Statistics

Values are presented as the mean ± SE. Comparisons between groups were performed by the Mann–Whitney *U* test using the Statview software (Abacus Concepts, Berkeley, CA, USA). To facilitate immunoblot and qPCR comparisons, we normalized the band density for the relative mRNA values by dividing them by the mean value for the SC group. Thus, the mean for the SC group was defined as 100%. *P* values of less than 0.05 were considered statistically significant.

## Results

### Results of the animal experiment

**Fig. 1:**
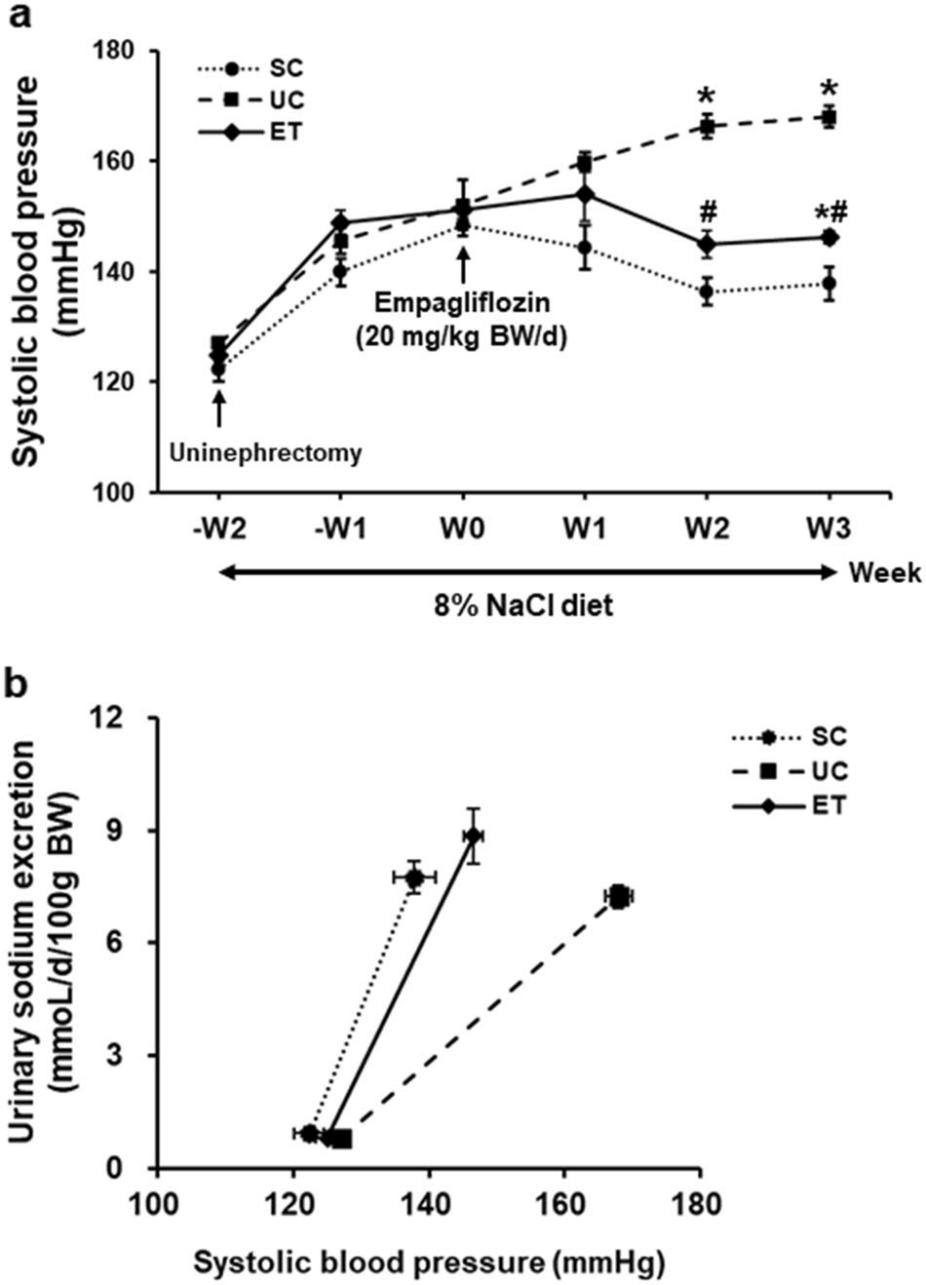
Comparisons of systolic blood pressure with empagliflozin treatment. All male Sprague-Dawley rats were fed a rodent diet with 8% NaCl for 5 weeks. To induce salt-sensitive hypertension, uninephrectomy was performed at the beginning. After 2 weeks of uninephrectomy, empagliflozin was orally administered for 3 weeks. Systolic blood pressure was measured weekly by a tail-cuff (**a**). Urinary sodium excretion and systolic blood pressure were compared in the context of the pressure–natriuresis relationship between the UC and ET rats (**b**). SCs, sham controls (*n* = 4); UCs, uninephrectomized controls (*n* = 4); ETs, empagliflozin-treated rats (*n* = 5). *P < 0.05 vs. SCs; ^#^*P* < 0.05 vs^.^ UCs by the Mann–Whitney *U* test

With high NaCl intake over 2 weeks, as shown in Fig. 1a, all rats showed a tendency for increased SBP until they were randomly divided into three groups, as follows: SC (*n* = 4), UC (*n* = 4), and ET rats (*n* = 5). When empagliflozin was administered for 3 weeks, SBP values were significantly different among the aforementioned three groups of animals. Whereas SBP in the SCs was mitigated (138 ± 3 mmHg), salt-sensitive hypertension was conversely enhanced in the UCs (168 ± 2 mmHg). The ETs had a significant decrease in SBP (146 ± 1 mmHg, *P* < 0.05) compared with that of the UCs. Figure 1b compares the PN relationship between the UC and ET rats. The right-shifted (impaired) PN relationship in the UCs was improved by empagliflozin treatment.

Table 2 shows plasma and urine data obtained at the end of the animal experiment. Urinary glucose excretion remarkably increased in the ETs, which is suggestive of the validity of our empagliflozin treatment. The ETs also demonstrated significant increases in urine output and urinary excretion of osmoles, compatible with osmotic diuresis. Accordingly, creatinine clearance significantly increased in the ET rats. However, the weekly measured urinary sodium excretions and fractional excretions of sodium were not significantly different between the groups. Compared with the SC and UC rats, urine protein was significantly increased in the ETs. Although statistically insignificant, albuminuria was induced in the UCs but ameliorated in the ETs over the 3-week empagliflozin treatment. Notably, urinary excretion of kidney injury molecule (KIM)-1 increased in the UCs but was significantly reversed in the ETs (Table 2). When KIM-1 messenger RNA (mRNA) was measured in the whole kidneys by qPCR analysis, it was found to be markedly increased in the UCs versus that in the SCs (640 ± 150% vs. 100 ± 17%, *P* *<* 0.05) and completely blocked in the ETs (116 ± 18%, *P* *<* 0.01).

**Table 2:** Functional parameters measured at the end of animal experiment

Parameters	SC (*n* = 4)	UC (*n* = 4)	ET (*n* = 5)
Body weight (g)	396 ± 6	409 ± 6	351 ± 11*^#^
Urine output (mL/d/100 g BW)	19.2 ± 0.6	23.0 ± 1.4*	42.0 ± 2.1*^#^
Plasma sodium (mmol/L)	146 ± 2	148 ± 2	148 ± 1
Plasma creatinine (mg/dL)	0.46 ± 0.05	0.52 ± 0.04	0.43 ± 0.05
Urine sodium excretion (mmol/d/100 g BW)	7.75 ± 0.88	7.24 ± 0.62	8.85 ± 1.46
Fractional excretion of sodium (%)	7.1 ± 0.8	6.7 ± 0.4	4.9 ± 0.2
Urine creatinine excretion (mg/d/100 g BW)	3.4 ± 0.2	3.8 ± 0.1	4.7 ± 0.3*^#^
Proteinuria (mg/d/100 g BW)	3.9 ± 0.5	4.2 ± 0.3	8.8 ± 0.3*^#^
Albuminuria (mg/d/100 g BW)	0.04 ± 0.01	0.23 ± 0.19	0.14 ± 0.05
Urine glucose excretion (mg/d/100 g BW)	0.5 ± 0.0	0.5 ± 0.0	470.2 ± 53.1*^#^
Urine osmoles (mosmoles/d/100 g BW)	20.6 ± 1.2	19.3 ± 0.7*	29.8 ± 1.8*^#^
Creatinine clearance (mL/min/100 g BW)	0.53 ± 0.05	0.51 ± 0.04	0.84 ± 0.04*^#^
Urine KIM-1/creatinine ratio (mg/μg)	2.79 ± 0.53	4.15 ± 1.44	1.49 ± 0.28*^#^

Values are presented as mean ± SE

*SC* sham controls, *UC* uninephrectomized controls, *ET* empagliflozin-treated rats

*P  <  0.05, versus SC; ^#^*P* <  0.05, versus UC

### Expression of HIF-1α and its related genes

**Fig. 2:**
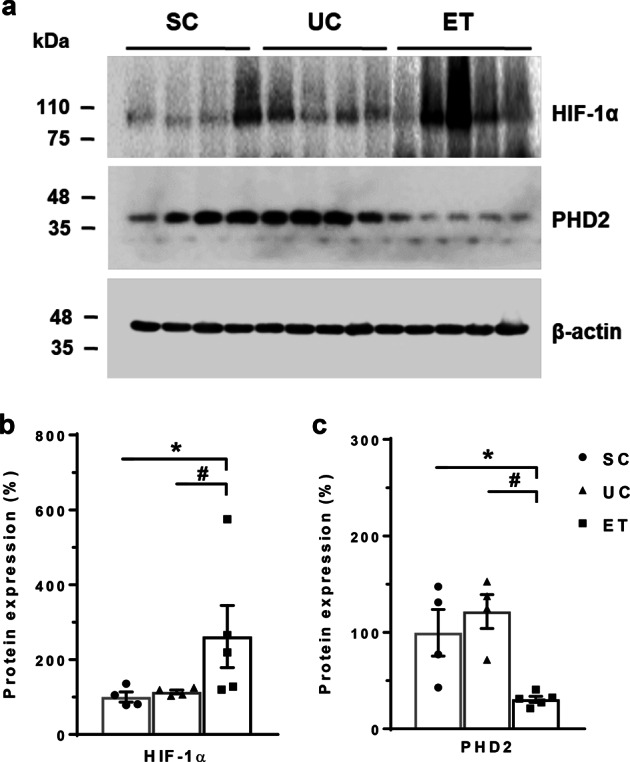
Immunoblot analysis of HIF-1α and HIF PHD2 in the renal medulla. Immunoblots with antibodies to HIF-1α and PHD2 are shown in the renal medulla (**a**). Each lane was loaded with a protein sample from a different rat. Densitometric analyses revealed a significant increase in HIF-1α (**b**) and a decrease in PHD2 (c) expression in the ETs versus the UCs. Anti-β-actin protein was used as a loading control. SCs, sham controls (*n* = 4); UCs, uninephrectomized controls (*n* = 4); ETs, empagliflozin-treated rats (*n* = 5). *P < 0.05 vs. SCs; ^#^*P* < 0.05 vs^.^ UCs by the Mann–Whitney *U* test

**Fig. 3:**
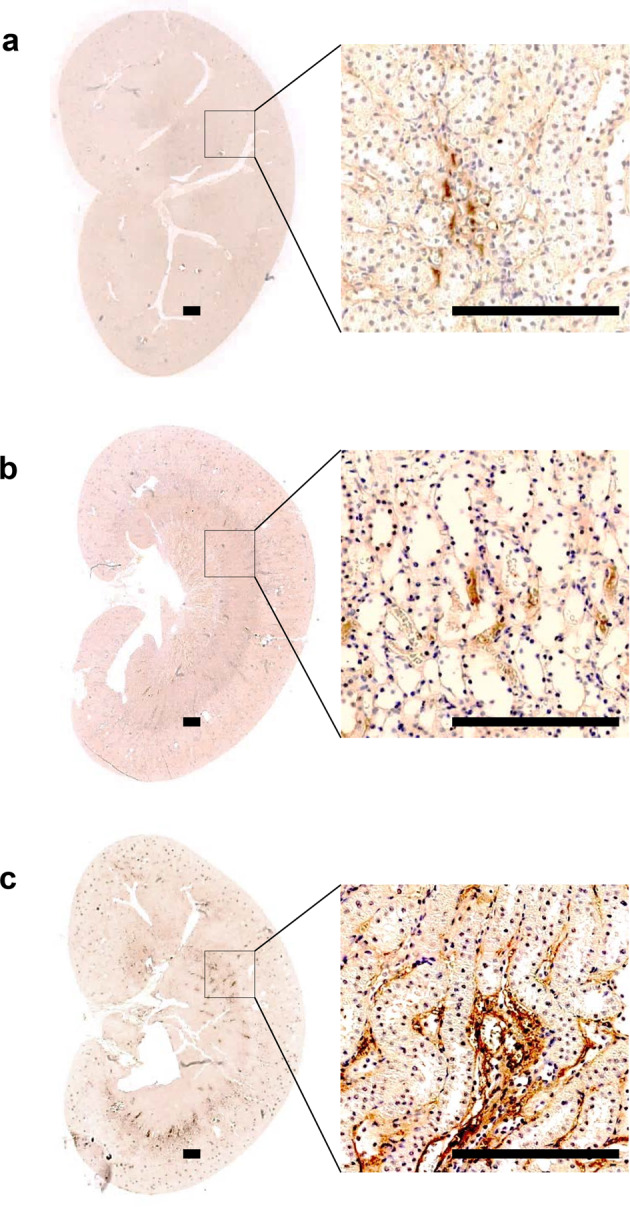
Immunohistochemical analysis of HIF-1α in rat kidneys. Representative images and corresponding enlargements of the boxed areas are shown for each group. Scale bar = 300 μm. In comparison with the SCs (a) and UCs (b), immunostaining for HIF-1α was stronger in the renal medulla of the ETs (c). SCs, sham controls (*n* = 4); UCs, uninephrectomized controls (*n* = 4); ETs, empagliflozin-treated rats (*n* = 5)

Figure 2 shows the results of immunoblot analysis of HIF-1α and PHD2 from renal medulla. Compared with the SCs (100 ± 13%), the UCs had no significant change in HIF-1α expression (114 ± 5%). However, ETs showed a significant increase in HIF-1α expression (262 ± 83%, *P* < 0.05) in comparison with levels in both the SCs and UCs. Consistent with this, PHD2 protein expression was significantly decreased in the ETs (31 ± 3%, *P* < 0.05) compared with that in both the SCs (100 ± 24%) and UCs (122 ± 18%). Figure 3 shows that immunostaining for HIF-1α was stronger in the renal medulla of the ETs than staining in the SCs and UCs.

**Fig. 4:**
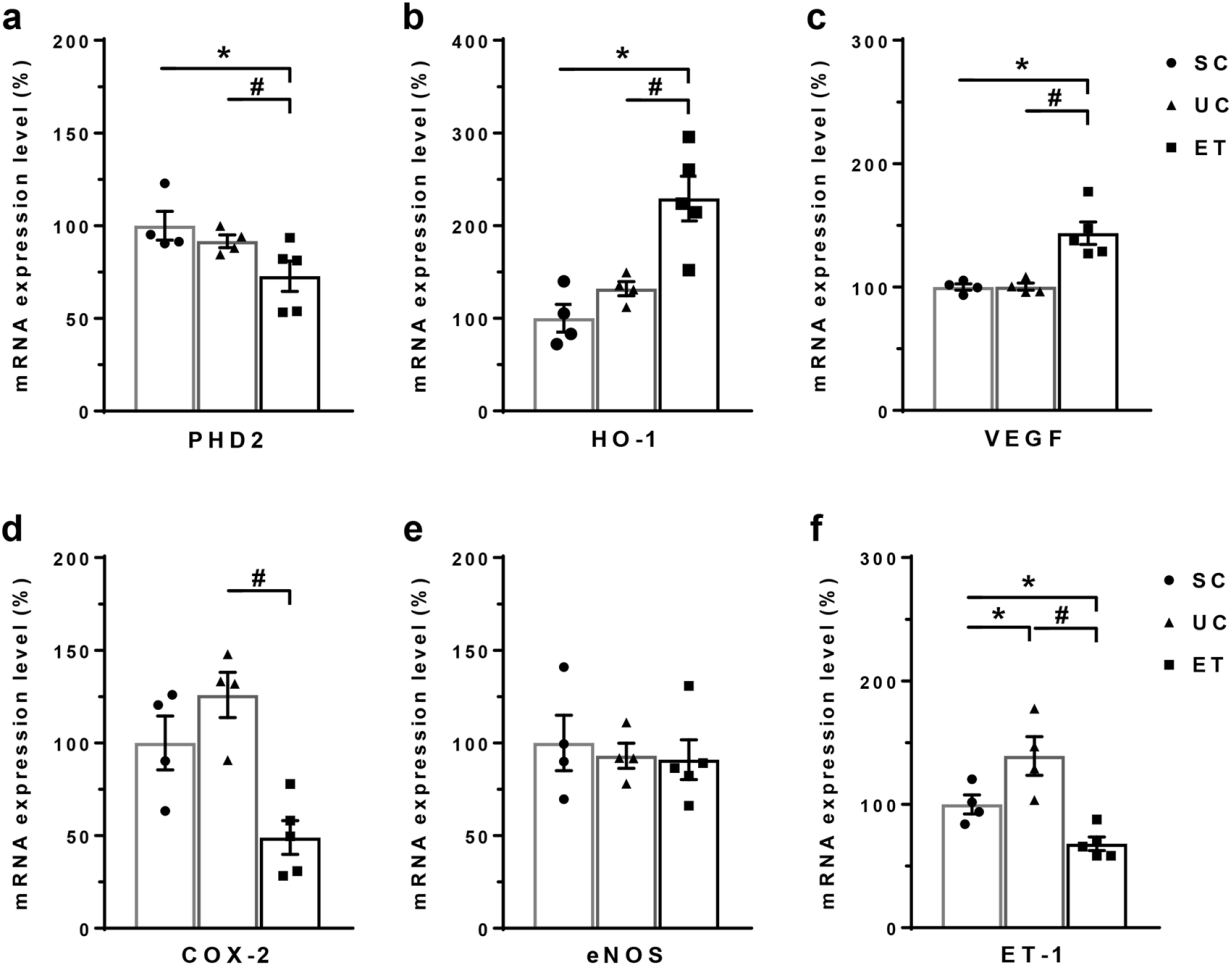
Quantitative polymerase chain reaction (qPCR) data for the mRNA levels of HIF-1α-related genes. qPCR analysis results for HIF-1α-related genes from whole kidneys are shown. GAPDH was used as an internal standard, and the relative amounts of target-mRNA are expressed as a percentage of the GAPDH mRNA level. PHD2, HIF prolyl-hydroxylase-2; HO-1, heme oxygenase-1; VEGF, vascular endothelial growth factor; COX-2, cyclooxygenase-2; eNOS, endothelial NOS; ET-1, endothelin-1; SCs, sham controls (*n* = 4); UCs, uninephrectomized controls (*n* = 4); ETs, empagliflozin-treated rats (*n* = 5). *P < 0.05 vs. SCs; ^#^*P* < 0.05 vs. UCs by the Mann–Whitney *U* test

Figure 4 summarizes the qPCR analysis data for HIF-1α-related genes from whole kidneys. Consistent with the upregulation of HIF-1α, PHD2 mRNA was significantly decreased in the ETs (73 ± 8%, *P* < 0.05) versus that in both the SCs (100 ± 8%) and UCs (92 ± 3%). In the ETs, the target genes heme oxygenase-1 (229 ± 24%, *P* < 0.05) and vascular endothelial growth factor (144 ± 9%, *P* < 0.05) mRNA were significantly increased. However, cyclooxygenase-2 mRNA was significantly decreased in the ETs (49 ± 9%, *P* < 0.05) compared with that in the UCs (126 ± 12%). In comparison with the SCs (100 ± 8%), endothelin-1 mRNA was significantly upregulated in the UCs (139 ± 16%, *P* < 0.05) but markedly reversed in the ETs (68 ± 5%, *P* < 0.05).

### Biomarkers of renal inflammation

**Fig. 5:**
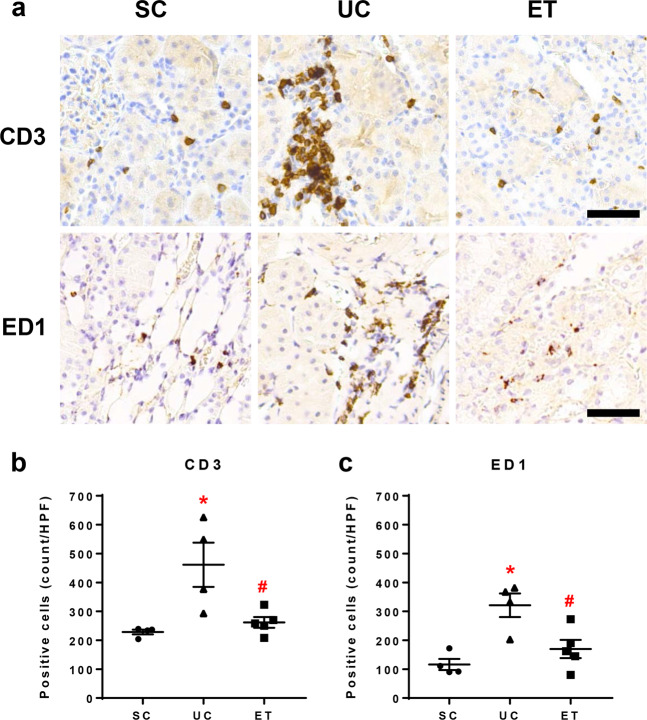
Immunohistochemical staining for CD3 and ED1 in the renal medulla. Images of representative tissue sections (a) and bar graphs of the number of positively stained cells in the interstitium (b and c) are shown. Scale bar = 100 μm. SCs, sham controls (*n* = 4); UCs, uninephrectomized controls (*n* = 4); ETs, empagliflozin-treated rats (*n* = 5). *P < 0.05 vs. SCs; ^#^*P* < 0.05 vs. UCs by the Mann–Whitney *U* test

Figure 5 shows the results of immunohistochemical staining of medullary sections for CD3 and ED1. Interstitial infiltration of CD3-positive T lymphocytes (461 ± 77 vs. 229 ± 8 cells/HPF, *P* < 0.05) and ED1-positive macrophages (321 ± 41 vs. 116 ± 19 cells/HPF, *P* < 0.05) was significantly increased in the UCs versus that in the SCs. Notably, compared with the UCs, the ETs had significantly fewer interstitial CD3-positive T lymphocytes (162 ± 19 cells/HPF, *P* < 0.05) and ED1-positive macrophages (170 ± 31 cells/HPF, *P* < 0.05).

**Fig. 6:**
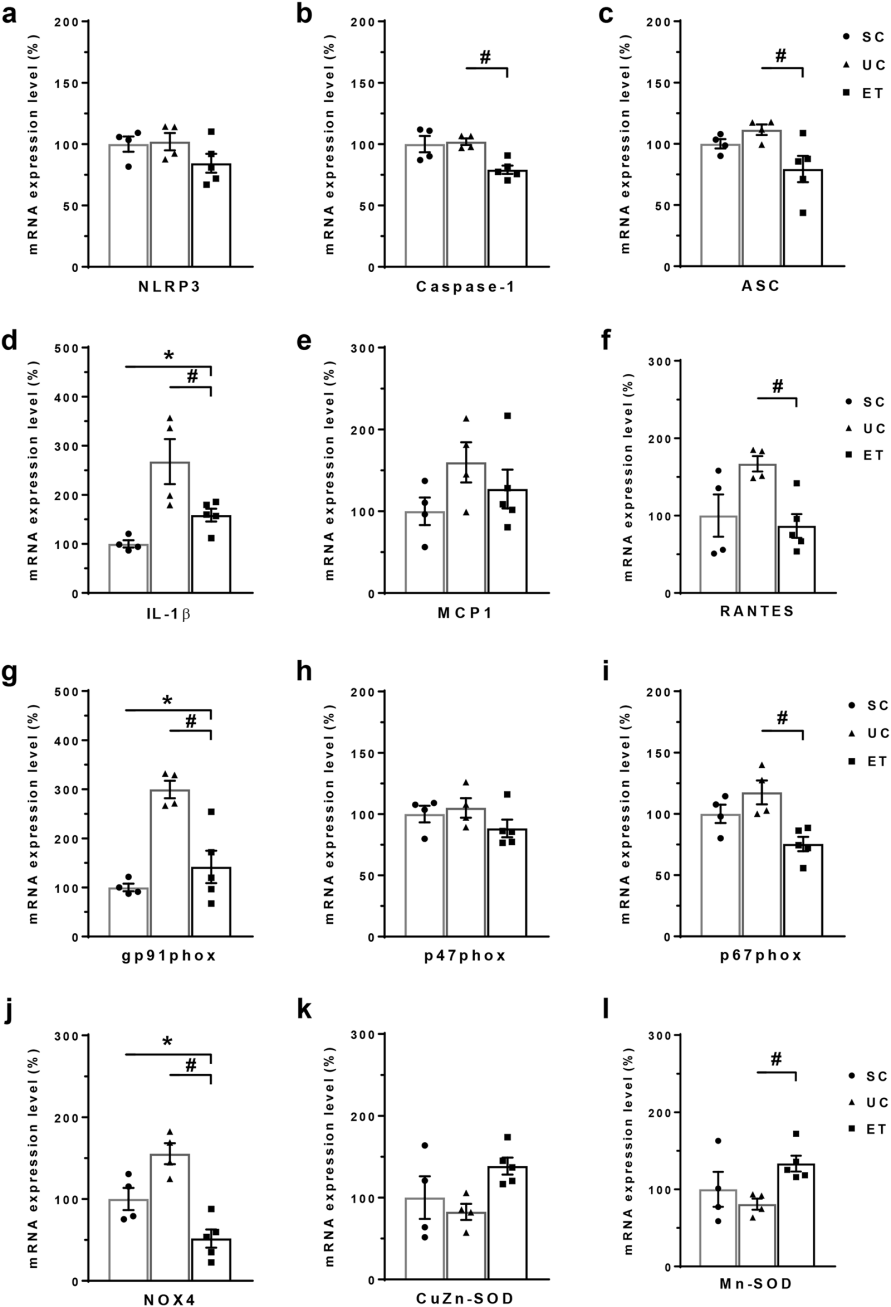
Quantitative polymerase chain reaction (qPCR) data for mRNA levels of inflammatory mediators and oxidative stress-related biomarkers. qPCR results for the inflammatory mediators and oxidative stress-related biomarkers are shown. GAPDH was used as an internal standard, and the relative amounts of target-mRNA are expressed as a percentage of the GAPDH mRNA level. NLRP3, NOD-like receptor family, pyrin domain-containing 3; caspase-1, interleukin 1β-converting enzyme (ICE); ASC, apoptosis-associated speck-like protein containing a caspase activation and recruitment domain; IL, interleukin; MCP1, monocyte chemotactic protein-1; RANTES, regulated on activation, normal T-cell expressed and secreted; NOX, nicotinamide adenine dinucleotide phosphate oxidase; CuZn-SOD, intracellular superoxide dismutase; Mn-SOD, mitochondrial SOD. SCs, sham controls (*n* = 4); UCs, uninephrectomized controls (*n* = 4); ETs, empagliflozin-treated rats (*n* = 5). *P < 0.05 vs. SCs; ^#^*P* < 0.05 vs^.^ UCs by the Mann–Whitney *U* test

Figure 6 summarizes the qPCR analysis data for the genes of the NLRP3 inflammasome, inflammatory cytokines, and nicotinamide adenine dinucleotide phosphate oxidase (NOX) from whole kidneys. In comparison with UCs, ETs had significantly lower mRNA levels of caspase-1, ASC, interleukin 1β, and RANTES. Similarly, ET rats had significantly lower mRNA levels of NOX2 components (gp91^phox^ and p67^phox^) and NOX4 versus levels in UC rats. Superoxide dismutases (SODs), including CuZn-SOD and Mn-SOD, showed an increasing tendency according to empagliflozin treatment.

## Discussion

In the present study, we demonstrated that SGLT2 inhibition was effective at controlling salt-sensitive hypertension, which was induced by renal mass reduction. When urinary sodium excretion was plotted against SBP, the PN relationship was impaired in our salt-sensitive hypertension model and partially corrected by empagliflozin treatment. Upregulation of renal medullary HIF-1α and the amelioration of renal inflammation and oxidative stress are plausible mechanisms by which salt sensitivity is relieved by empagliflozin treatment.

Whereas the usefulness of SGLT2 inhibitors as antidiabetic agents is now established, the role of SGLT2 inhibitors in the treatment of nondiabetic kidney disease is just emerging [15]. Because salt sensitivity increases as functioning renal mass decreases [13], we successfully induced salt-sensitive hypertension by high NaCl intake and uninephrectomy. Although the blood-pressure-lowering effects of SGLT2 inhibitors are clearly known in diabetic patients and animals [16, 17], whether these are similarly exerted actions in nondiabetic animals or patients is unknown. A recent pilot study reported that dapagliflozin treatment decreased SBP in subtotally nephrectomized rats but not in patients with focal segmental glomerulosclerosis [18].

The potential mechanisms of the blood pressure decrease caused by SGLT2 inhibition in diabetes were postulated to be improved glucose control, weight loss, volume contraction due to osmotic diuresis, and improved arterial stiffness [19]. However, these mechanisms remain elusive in nondiabetes. In this study, the PN relationship was investigated to identify the effects of SGLT2 inhibition. Figure 1b clearly shows that salt sensitivity was improved by empagliflozin treatment.

In this study, sodium excretion did not increase in empagliflozin-treated rats compared with excretion in uninephrectomized controls when measured weekly. According to previous studies, the natriuretic response to SGLT2 inhibitors is modest and transient [20, 21]. Thus, the initial natriuretic response might have been unnoticed by us. On the other hand, osmotic diuresis associated with remarkable glycosuria was so evident that it could enhance creatinine clearance. The latter finding is contradictory to the known effect of SGLT2 inhibition on tubuloglomerular feedback [4] and appears to be related to our animal model (uninephrectomy + high salt intake). Body weight is another factor that influences blood pressure. Obesity increases renal sodium reabsorption and impairs PN by activation of the renin–angiotensin and sympathetic nervous systems and by altered intrarenal physical forces [22]. In our study, empagliflozin-treated rats had a lesser degree of body weight gain than did the uninephrectomized controls (122 ± 12 vs. 70 ± 12 g, *P* = 0.05) over 3 weeks.

Renal medullary circulation plays an important role in PN by influencing tubular sodium reabsorption [23]. Among the factors that exhibit a profound impact on renal medullary blood flow, we paid attention to HIF-1α because local oxygen consumption may be a determinant of blood flow in the renal medulla [24]. HIF-1α is more abundantly expressed in the renal medulla than that in the cortex and is modulated in the renal medulla by changes in the partial pressure of oxygen (PO_2_) [25]. Li et al. showed that salt-sensitive hypertension was induced when HIF-1α decoy oligodeoxynucleotides were transfected into the renal medulla in uninephrectomized Sprague-Dawley rats, and they concluded that HIF-1α activation participates in the regulation of renal medullary circulation and long-term arterial blood pressure [26].

In this study, we showed that HIF-1α was activated by empagliflozin treatment. The expression of HIF-1α protein was increased in the renal medulla, while PHD2 protein and mRNA were decreased in rat kidneys with empagliflozin treatment. We postulated that renal medullary hypoxia might be provoked by the use of SGLT2 inhibitors because the tubular workload is shifted from the cortical proximal tubule to the medullary thick ascending limb. This may explain why we observed an increase in total proteinuria without an accompanying change in albuminuria. It has been reported that medullary PO_2_ was reduced in both control and diabetic kidneys when SGLT was inhibited in rats [27]. A recent study employing computational rat kidney models showed that SGLT2 inhibition was accompanied by a shift in oxygen-consuming active transport to the outer medulla in a kidney, along with reduced nephron numbers [28]. In addition, HIF-1 was induced when dapagliflozin treatment was given to hypoxic HK2 cells and mice with ischemia–reperfusion injury [29].

Although the proximal tubule in the cortex is the major site of tubular proteinuria, the excretion of exosomes is another source of proteinuria [30]. The origin of urinary exosomes may include all of the epithelial cells in the nephron, and Tamm–Horsfall protein (THP), an apically expressed, glycosylphosphatidylinositol-linked protein expressed in the thick ascending limb of Henle, is readily identifiable in urine [30]. Notably, previous studies have shown that overt proteinuria can be induced by medullary tubular injury. In isolated perfused rat kidneys, Shiga toxin significantly increased urinary protein excretion (from 61 ± 23 to 169 ± 28 μg/min, *P* < 0.01) in association with the injury to the medullary thick ascending limbs [31]. Remarkable proteinuria (78 ± 12 versus 17 ± 2 mg/day; *P* < 0.01) was produced in conscious rats when renal medullary hypoxia was induced by nitric oxide synthase inhibition [32]. Furthermore, the increase in urinary THP was demonstrated in streptozotocin diabetic rats with damage to the thick ascending limb of the loop of Henle [33]. When these reports are taken together, medullary hypoxia might be linked to increased proteinuria.

We also tested the downstream pathways from HIF-1α activation. Previous studies have shown that in response to salt loading, the activation of endothelin-1, nitric oxide, and cyclooxygenase-2 contributes to natriuresis [34–37]. In this study, the mRNA expression of heme oxygenase-1 and vascular endothelial growth factor were increased by empagliflozin treatment. However, the mRNA expression of endothelin-1 and cyclooxygenase-2 was decreased by empagliflozin treatment. The latter responses appear to be HIF-1α-independent.

The altered PN relationship may result from renal inflammation [38]. Thus, the treatment of renal inflammation could be connected with the relief of salt-sensitive hypertension. In this study, interstitial infiltration of inflammatory cells in the salt-loaded solitary kidneys was improved by empagliflozin treatment. The urinary KIM-1 protein and renal KIM-1 mRNA were increased in our salt-sensitive hypertension model and reversed by empagliflozin treatment, which is compatible with the renoprotective effect of SGLT2 inhibition. In Dahl salt-sensitive rats, high NaCl intake induced activation of the NLRP3 inflammasome in the renal medulla [39]. We showed that the upregulation of interleukin-1β in our salt-sensitive hypertension model was blocked by empagliflozin treatment.

As an important source of inflammation, renal reactive oxygen species and NOX may cause salt-sensitive hypertension [40]. We showed that the upregulation of NOX2 and NOX4 in our salt-sensitive hypertension model was blocked by empagliflozin treatment. These results were associated with antioxidant defense mechanisms. Previous studies on the anti‐inflammatory effects of SGLT2 inhibitors have been limited to experimental models of diabetic kidney disease [41].

In conclusion, nondiabetic salt-sensitive hypertension was improved by empagliflozin treatment via the upregulation of renal medullary HIF-1α and the amelioration of renal inflammation and oxidative stress. The upregulation of HIF-1α may contribute to the protective effects of SGLT2 inhibitors on blood pressure, and empagliflozin exerts anti-inflammatory action in nondiabetic kidney disease as well.
